# (Very) Small Stem-like Cells in Human Cell Cultures

**DOI:** 10.3390/cancers15235520

**Published:** 2023-11-22

**Authors:** Jan Jakub Lica, Bhaskar Pradhan

**Affiliations:** 1 Department of Regenerative Medicine, Faculty of Medicine, Medical University of Warsaw, 02-097 Warsaw, Poland; 2 Department of Molecular Biochemistry, Faculty of Chemistry, University of Gdansk, 80-308 Gdansk, Poland; 3 Department of Biochemistry, Faculty of Pharmacy, Medical University of Warsaw, 02-097 Warsaw, Poland; bhaskar.pradhan@wum.edu.pl

**Keywords:** cell cultures: A549, HEK293, HL-60, Jurkat, KG1a, K562, and Raji, cancer stem-like cells, leukemic stem-like cells, very small cancer stem-like cells, very small leukemic stem-like cells, very small embryonic-like stem cells, cluster of differentiation: CD34, CD45, CD56, and CD2

## Abstract

**Simple Summary:**

VSELSCs are considered the Holy Grail of regenerative medicine. The published transformations from VSELSCs to VSCSCs to CSCs have prompted us to re-analyze the results of previous research and conduct new studies on the existence of very small stem-like cells in continuously growing cell lines. Utilizing well-established cell cultures with varying levels of primitive cell content, specific CD markers for VSELSCs, side and forward scattering, DNA content analysis, and functional studies, a cytological method for detecting this cell stage has been developed.

**Abstract:**

Very Small Embryonic-like Stem Cells (VSELSCs) and Very Small Cancer Stem Cells (VSCSCs) are fields of intensive research. Although the presence in vitro of VSELSC and VSCSC cellular stage analogs appear probable, it has yet to be published. Utilizing established human cell cultures with varying populations of primitive cells, stained with CD markers specific to primitive stages, in addition to a fluorescent DNA dye, and following histochemical processing, we have developed a cytological method for detecting Very Small Leukemic Stem-like Cells (VSLSLCs), Very Small Cancer Stem-like Cells (VSCSLCs), and VSELSCs. This detection provides an opportunity to advance research in these areas.

## 1. Introduction

Stem Cells (SCs) are a population of minimally differentiated primitive cell stages within an organism [1]. However, when these cells are cultured in vitro, due to epigenetic modifications and dysfunctions, a more appropriate term for them is Stem-like Cells (SLCs) [2,3,4,5,6,7]. In cancer, a population of CSCs was also observed, and these stages that exist outside the organism are named Cancer Stem-like Cells (CSLCs) [8]. Both CSCs and CSLCs represent malignant cells of epithelial origin, while malignancies originating from other germ layers have distinct designations, e.g., leukemia has its specific Leukemic Stem Cells (LSCs) [9]. The study of SCs involves investigations into their precursor developmental stages—VSELSCs [10]; however, these cellular stages are still controversial (Supplementary Text ST1). Interestingly, VSELSCs have also been found in the context of cancer [11,12,13,14]. Although there is information regarding CSLCs and LSLCs, reports addressing (V)SCSLCs, VSLSLCs, and VSELSCs in vitro are notably lacking [8,15,16].

Our earlier study revealed a phenomenon that was previously observed in healthy human hematopoietic cells: the enrichment of culture with primitive stages through the control of cell density [15,16,17]. The LSLCs identified in HL-60 by our team displayed an average size of approximately 9–12 µm [15,16] and were commonly situated in regions C1–C4 and D1–D4 on scatter dot plots (Supplementary Figures S1 and S2), in alignment with findings reported by other researchers [8]. Studies detailing the morphology of human VSELSCs have reported their size to be around 5–7 µm [10]. Potential VSLSLCs were expected to possess a similar size that can increase during developmental stage transformations, reaching approximately twice the size under self-renewal or forming the LSLCs [15]. Corresponding morphological features of these stage transformations should be visible in scatter dot plots, representing the resting phase (which may be about 5–7 µm), symmetric division (up to 10–14 µm, but it might also be 5–7 µm), and asymmetric division (leading the developmental stages to an increase in size). We cannot exclude other asymmetric divisions occurring at cell sizes of 5–7 µm, leading to the formation of smaller and less-differentiated cells than LSLCs. Because of all the possibilities of morphological similarity between hypothetical developmental stages, we decided to divide the scatter dot plot region of interest into two distinct events: VSLSLCs and Small Leukemic Stem-like Cells (SLSLCs). It is worth noting that SLSLCs might represent a developmental stage of VSLSLCs undergoing changes in DNA distribution. 

We hypothesize that (V)SLSLCs function as precursors to LSLCs, and their numbers in cell density-dependent HL-60 sublines, along with other developmental stages, should exhibit differences [15,16]. Considering the data regarding CD markers for VSELSCs, we hypothesized that CD34 would potentially be among the first markers to be observed on the cell surface. However, CD45, as a negative marker for VSELSCs, is commonly used by researchers [18,19,20,21,22], as well as in Euro Flow standardization dedicated to hematological probes [23,24,25]. CD markers exclusively targeting VSELSCs, such as NANOG/CD44, SSEA4, and CD133, despite their effectiveness for samples directly from the organism [18,21], show lower efficiency in cell lines [19,20]. To ensure consistency in interpreting findings from previous studies [15,16] and to distinguish between different developmental stages within the tested cell lines, we chose CD56 [26,27,28]. Its presence also serves as an indicator of a poorer prognosis in multiple myeloma [27]. For ALL cells (Jurkat and Raji), the CD56 marker was substituted with CD2 [29]. To confirm the presence of DNA and its degree of condensation in (V)SLSLCs events, we examined the CD34 marker level in comparison to DNA content in KG1a. For more detailed information on (V)SLSLCs data from previous work on HL-60 [15,16], please refer to Supplementary Figures S1 and S2 and their descriptions.

## 2. Materials and Methods

### 2.1. Drugs

APC Mouse Anti-Human CD34 (555824, BD Pharmanigen, San Diego, CA, USA), PE Mouse Anti-Human CD45 (555483, BD Pharmanigen, USA), FITC Mouse Anti-Human CD56 (cat: 345811, BD Pharmanigen, USA), FITC Mouse Anti-Human CD2 (555326, BD Pharmanigen, USA), FcR Blocking Reagent, human (130-059-901, Miltenyi Biotec, North Rhine-Westphalia, Germany), Hoechst 33342 (H21492, Thermo Fisher Scientific, Waltham, MA, USA), Trypan Blue (T8154, Merck, Darmstadt, Germany), RPMI 1640 with L-glutamine (10-040-CV, Corning, Corning, NY, USA), and Fetal Bovine Serum (35-079-CV, Corning, USA).

### 2.2. Cell Cultures

All cell lines originated from the ATCC collection. More detailed information about the culture conditions of HL-60, A549, and HEK293 can be found in [15,16]. The Raji, Jurkat, KG1a, and K562 cell lines were cultured in RPMI-1640 medium with L-glutamine (10-040-CV, Corning, USA) and supplemented with 10% (20% for KG1a) Fetal Bovine Serum (35-079-CV, Corning, USA) and the antibiotics penicillin and streptomycin at 37 °C in a humidified atmosphere with 5% CO_2_ and 95% air.

### 2.3. Cell Surface Staining

Staining was performed according to Yale Medicine School Flow (FACS) protocol https://medicine.yale.edu/immuno/flowcore/protocols/analysis/ (accessed on March 2023). Briefly, cells from the culture, with viability not less than 95% as measured by the Automated Brightfield Cell Counter LUNA-II™ (Logos Biosystems, Anyang-si, Republic of Korea), or Hemocytometer using Trypan Blue (T8154, Merck, Germany), were centrifuged at 1500 rpm for 5 min. They were then re-suspended in 100 µL of 10% Fetal Bovine Serum (35-079-CV, Corning, USA) in PBS. Next, 100 µL of FcR Blocking Reagent, human (130-059-901, Miltenyi Biotec, Germany), diluted at a 1:50 ratio in 10% Fetal Bovine Serum (35-079-CV, Corning, USA) in PBS, was added and incubated for 20 min. The samples were subsequently centrifuged at 1500 rpm for 5 min, and the supernatant was discarded. Next, 20 µL of the CD anti-body was added and incubated for 30 min. The samples were washed 1–3 times in 10% Fetal Bovine Serum (35-079-CV, Corning, USA) in PBS (centrifuged at 1500 rpm for 5 min each time) and re-suspended in 200 µL to 1 mL. The samples were read using the FACSVerse flow cytometer (BD Bioscience, Franklin Lakes, NJ, USA). Flow cytometry results were analyzed with the free online software Floreada.io (https://floreada.io/ (accessed in November 2023; the last update was carried out in June 2023)).

### 2.4. DNA Content vs. CD34

The CD34 staining procedure APC Mouse Anti-Human CD34 (555824, BD Pharmanigen, USA) was conducted following the previously mentioned protocol. Hoechst 33342 (H21492, Thermo Fisher Scientific, USA) was introduced to the sample 5 min prior to the sample read. Subsequently, the samples underwent a single wash and later re-suspended in PBS (centrifuged at 1500 rpm for 5 min) before being read by FACSVerse flow cytometer (BD Bioscience, USA). The outcomes of the flow cytometry were analyzed with the free online software Floreada.io (https://floreada.io/ (accessed in November 2023; the last update was carried out in June 2023)).

### 2.5. Development of Primitive HL-60 and Primitive A549

To obtain a culture containing approximately zero LSLCs and CSLCs, the cell density of Primitive HL-60 was reduced to below 2.5 × 10^3^ cells per mL, and Primitive A549 was decreased to below 2.5 × 10^3^ cells per 175 cm^2^. Scatter dot plots illustrate the events of at least half of the A549 and HL-60 culture, and the rest was seeded in the new flask (353112, BD Falcon, Franklin Lakes, NJ, USA). Samples were read by Guava easyCyte (Merck-Millipore, Burlington, MA, USA). Flow results were analyzed with the free online software https://floreada.io/ (accessed in November 2023; the last update was carried out in June 2023).

## 3. Result

### 3.1. (Very) Small Leukemic Stem-like Cells in Myeloid Leukemia Cultures

**Figure 1:**
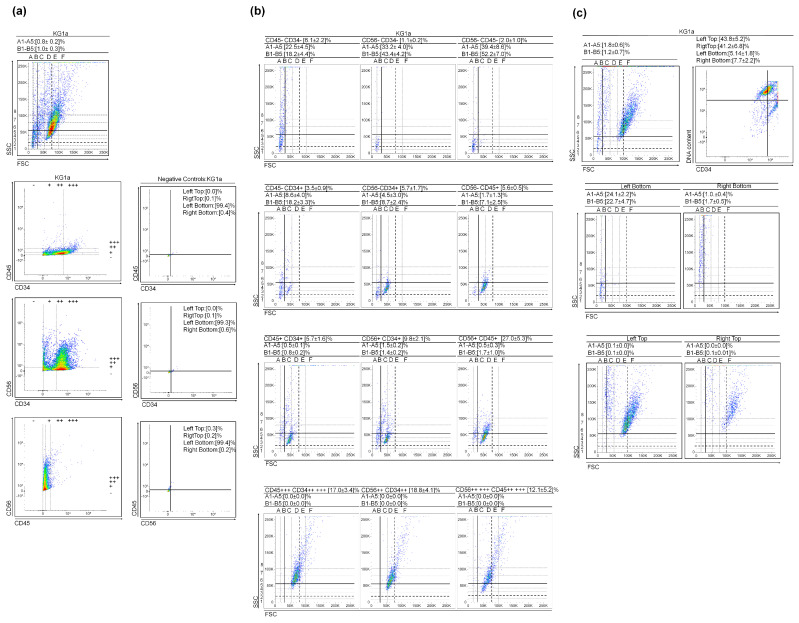
KG1a cytological parameters and cluster differentiation patterns. (**a**) Scatter dot plots of the cultures and CD45 vs. CD34, CD56 vs. CD34, and CD56 vs. CD45 gate regions. The negative controls obtained during the CD marker staining process provide representative data; (**b**) Selected scatter dot plots from gate regions; (**c**) DNA content (Hoechst 33342) vs. CD34 in KG1a. Values represent mean ± SD from five (**a**,**b**) and three (**c**) independent experiments.

**Figure 2:**
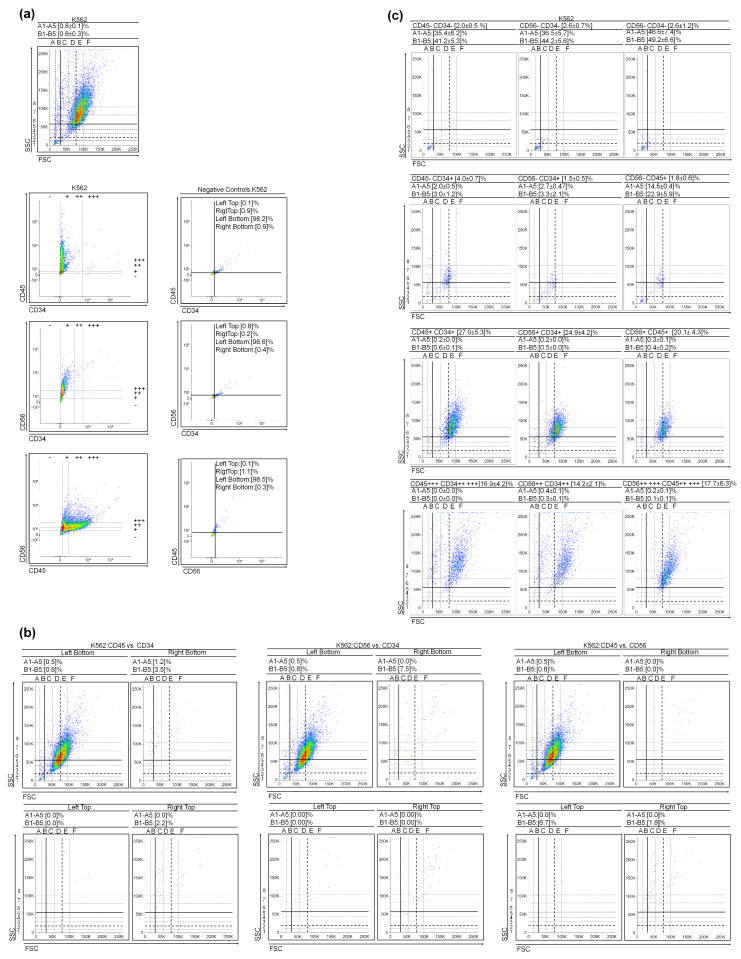
K562 cytological parameters and cluster differentiation patterns. (**a**) Scatter dot plots of the cultures and CD45 vs. CD34, CD56 vs. CD34, and CD56 vs. CD45 gate regions; (**b**) Selected scatter dot plots of negative controls obtained during CD marker staining, representative data; (**c**) Selected scatter dot plots from gate regions. Values represent mean ± SD from five independent experiments.

To determine if the presence of VSLSLCs and/or SLSLCs is not limited to HL-60 (Supplementary Figures S1 and S2), we analyzed the levels of CD34, CD45, and CD56 markers in two other myeloid leukemia cell cultures: KG1a—Acute Myeloid Leukemia (AML) and K562—Chronic Myeloid Leukemia (CML during blast crisis) (Figure 1 and Figure 2). Please refer to Figure 1b for KG1a and Figure 2c for K562, illustrating the selected scattering dot plots of these three CD markers. Comparable to HL-60 (Supplementary Figures S1 and S2), the regions corresponding to (V)SLSLCs in KG1a and K562 primarily exhibit negative events for CD34, CD45, and CD56 markers (Figure 1 and Figure 2). An increase in CD34+ (while maintaining CD45− and CD56−) markers is associated with a decrease in the counts in the (V)SLSLCs regions and an increase in the number of LSLCs. This suggests that the LSLCs in these cell lines might originate from (V)SLSLCs (Figure 1 and Figure 2). As the levels of CD markers continue to rise, there is a corresponding decline in the count of (V)SLSLCs. Furthermore, we conducted a DNA content vs. CD34 analysis for KG1a using Hoechst 33342 and the CD marker. The resulting scatter dot plots are presented in Figure 1c. The CD34− and CD34+ events were found to co-localize with DNA within the regions corresponding to (V)SLSLCs (Figure 1c). Importantly, the degree of chromatin condensation in these events is lower than what we observed in the apoptotic bodies (ABs).

### 3.2. (Very) Small Leukemic Stem-like Cells in Lymhoid Leukemia Cultures

**Figure 3:**
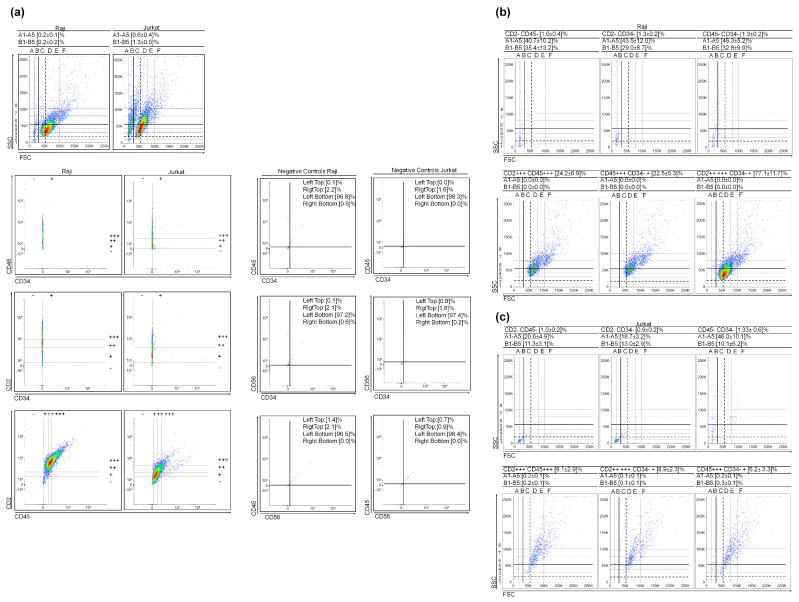
Raji and Jurkat cytological parameters and cluster differentiation patterns. (**a**) Scatter dot plots of the cultures and CD45 vs. CD34, CD2 vs. CD34, and CD2 vs. CD45 gate regions. The negative controls obtained during the CD marker staining process provide representative data. Selected scatter dot plots of negative controls are presented in Supplementary Figure S3; (**b**,**c**) Selected scatter dot plots from gate regions. Values represent mean ± SD from five (Raji) and three (Jurkat) independent experiments.

For verification of the presence of (V)SLSLCs, we analyzed the levels of CD34, CD45, and CD2 markers in two Acute Lymphoblastic Leukemia (ALL) sub-types: Raji and Jurkat (Figure 3a). The selected scatter dot plots from marker gates are presented in Figure 3b. Both ALL sub-types exhibit a low expression of the CD34 marker. Nevertheless, the results are similar to those observed in myeloid leukemia, indicating triple-negative events in the (V)SLSLC regions (Figure 3). It is noteworthy that the count of (V)SLSLCs on the scatter dot plot decreased as the CD marker levels increased.

### 3.3. Very Small Cancer Stem-like Cells in Non-Small Cell Lung Cancer

**Figure 4:**
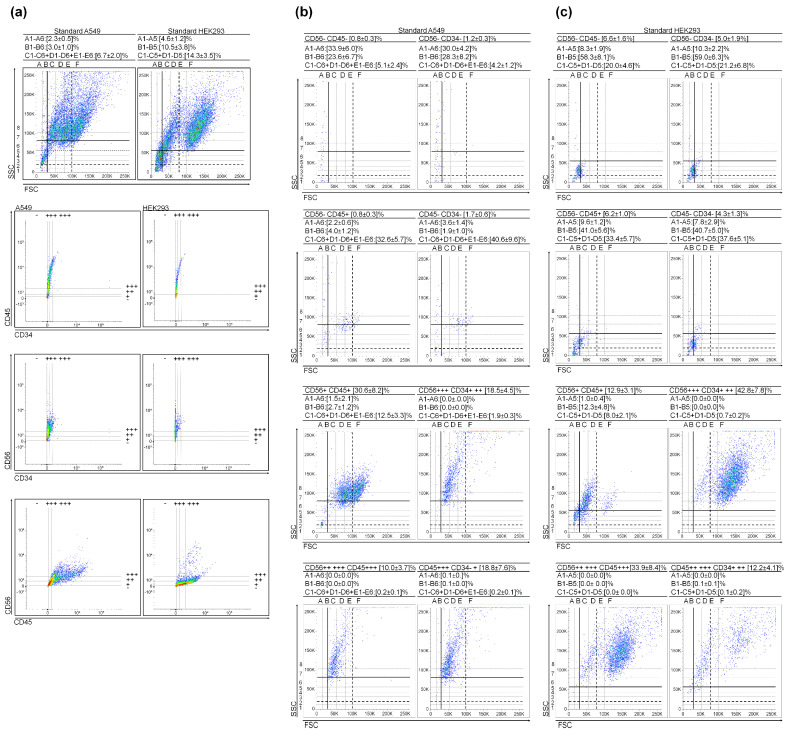
A549 and HEK293 cytological parameters and cluster differentiation patterns. (**a**) Scatter dot plots of the A549 and HEK293 cultures as well CD45 vs. CD34, CD56 vs. CD34, and CD56 vs. CD45 gate regions; (**b**) Selected scatter dot plots from A549 gate marker regions; (**c**) Selected scatter dot plots from HEK293 gate marker regions. Values represent mean ± SD from three independent experiments.

The CD34+ vs. CD45− immunophenotype is recognized for primitive hematopoietic stages and their developmental precursors, VSELSCs. Our aim was to determine whether these stages exist in cell cultures beyond leukemia. For this investigation, we examined the surface levels of CD34, CD45, and CD56 markers (see Figure 4a), and the selected scatter dot plots from marker gates are presented in Figure 4b. The results are analogous to those obtained for leukemias. The regions associated with (V)SCSLCs displayed mostly triple-negative CD events or CD34+, CD45−, and CD56− (Figure 4). Consistent with earlier observations, an increase in the surface expression from CD34− to CD34+ at CD45− and CD56− led to a reduction of approximately 10-fold in (V)SCSLC events and a 6-fold reduction in SCSLC events. At the same time, there was a 6-fold increase in the number of events in the CSLC regions (Figure 4). An increase in the levels of CD markers is associated with a rise in the mean values of granularity and size. Additionally, in Supplementary Figure S4a, we present cell density-dependent changes in cytological parameters (including VSCSLCs number) of A549 cultures, as previously published in [16].

### 3.4. Very Small Embryonic-like Stem Cells in Embryonic Kidney Cell Culture

Finally, we explored the presence of (V)SELSCs in HEK293, derived from embryonic kidney (Figure 4a,c). We examined the surface levels of CD34, CD45, and CD56 markers (Figure 4a), as depicted in the scatter dot plot patterns shown in Figure 4c. Similar to previous findings, the regions associated with (V)SELSCs were predominantly characterized by triple CD marker-negative events (Figure 4c). As the CD34 marker level increased from—to + (while CD45− and CD56−), there was an approximate 2-fold increase in SLCs, accompanied by a decrease in (V)SELSCs (Figure 4). It is worth noting that this cell line exhibited the highest proportion of triple-negative CD events. Additionally, the scatter dot plots in Supplementary Figure S4b provide insights into how the cytological characteristics of HEK293, including the count of (V)SELSCs, depend on the response to controlling cell density. This phenomenon has been previously discussed in [16].

### 3.5. Exploring the Functionality of (V)SCSLCs and (V)SLSLCs

**Figure 5:**
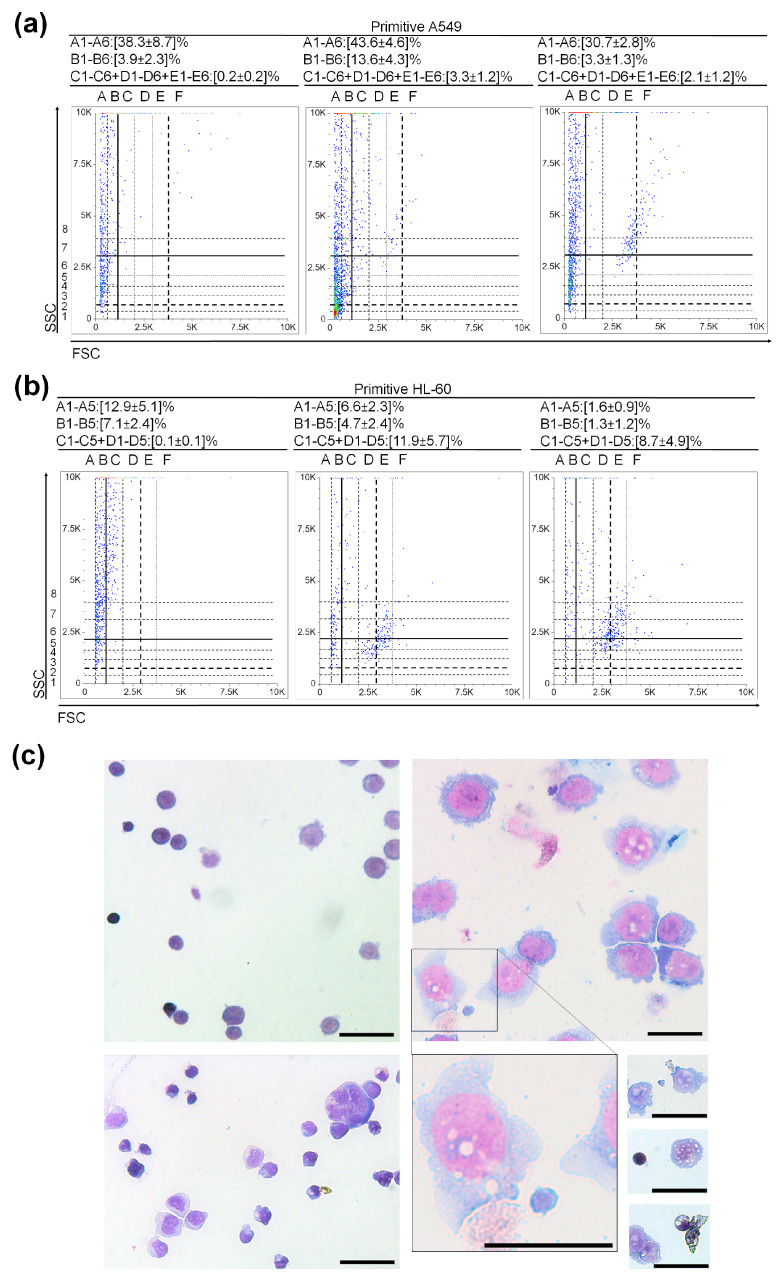
Functionality of A549 and HL-60: (**a**) Initial scatter dot plots of the established Primitive A549; (**b**) Initial scatter dot plots of the established Primitive HL-60; (**c**) Micro-photographs of Primitive HL-60 that re-grew from a culture with an initial number of LSLC events reaching 0.1%. Given the extremely limited number of events that initiated the Primitive HL-60 and considering that at least 50% of the culture volume was used for flow cytometry data acquisition, along with accounting for event loss during May–Grunwald–Giemsa (MGG) staining, we were unable to capture photographs of the very early stages of Primitive HL-60. Micro-photograph of early developmental stages of Primitive HL-60 (upper left panel). Micro-photograph capturing blast-like and CFU-like developmental stages, with a square outline highlighting an event exhibiting morphological features of VSLSLCs (size, strongly alkaline cytoplasm, condensed chromatin lower than in apoptotic bodies (ABs)) (upper right panel). Images presenting early developmental cells and colonies of Primitive HL-60 (lower left panel). An enlarged micro-photograph depicting a potential VSLSLC. Images of extra-cellular vesicles (EVs), an event with highly condensed chromatin that might be ABs or cells, and a rare event illustrating the early stages of HL-60 colony development (lower right panel). These study results align with those obtained in earlier research when establishing anthracycline cross-resistant HL-60 [15]. For MGG stains, cells were cytospun on a glass slide (850 RPM, 4 min) using Cytofuge 2 StatSpin (HemoCue Inc., Brea, CA, USA). Micro-photograph was taken by an Olympus BX60 (Olympus, Tokyo, Japan) microscope coupled to XC50 CCD camera, Scale bar: 25 µm. Values (**a**,**b**) represent mean ± SD from five independent experiments.

In our approach to evaluate functionality, we utilized the accuracy of flow cytometer while minimizing stress on cells during staining, measurement, and clonogenicity tests. You can find more details in Supplementary Text ST2. This approach focuses on cytometric monitoring and analysis of primary A549 and HL-60 cultures maintained at extremely low cell densities. A substantial decrease in cell density within the primitive culture led to an initial population characterized by a pattern featuring numerous (V)SCSLCs and (V)SLSLCs, with an almost negligible presence of CSLCs and LSLCs (Figure 5). Reducing the Primitive A549 culture density to below 2.5 × 10^3^ cells per 175 cm^2^ resulted in a decline in CSLCs reaching 0.0 (Figure 5a). The scatter dot plots presented in Figure 5a illustrate the change in the ratio of (V)SCSLC and CSLC events at the establishment of Primitive A549. During the formation of CSLCs, the number of (V)SCSLCs decreases (Figure 5a). Lowering the Primitive HL-60 density below 2.5 × 10^3^ cells per mL results in a decrease in LSLCs, reaching 0.1% (Figure 5b). The scatter dot plots illustrating the re-growth of Primitive HL-60 are presented in Figure 5b, displaying an approximately 8-fold decrease in the ratio of VSLSLCs and a 9-fold increase in LSLCs. We successfully replicated the experiment’s results independently five times for both Primitive A549 and Primitive HL-60. Furthermore, for Primitive HL-60 established from (V)SLSLC events, we included micro-photographs of MGG-stained cells in Figure 5c. These images exhibit features showing that Primitive HL-60 contains different types of primitive stages, e.g., megakaryocytic-like and erythroid-like (Figure 5c).

## 4. Discussion

Even when primitive cells are exposed to changes caused by adapting to a new environment, such as a culture flask, LSLCs are able to preserve their cytological and physiological characteristics [9,15]. Over time, LSCs also accumulate mutations, and relying solely on these problems for distinguishing between CSCs and LSCs with CSLCs, LSLCs become less effective. Using CSLCs, LSLC (very) small precursors as an alternative model, might improve the accuracy of identifying and targeting their counterparts within the organism for elimination.

Previously, we demonstrated the feedback mechanisms in primitive stages of human leukemic, cancer, and non-cancer cultures [15,16] These results correspond with those obtained for human hematopoietic cell cultures by other researchers [17]. Observed correlations between the developmental stages of VSELSCs–VSCSCs–CSCs [11,12,13] led us to investigate the data for the presence of VSLSLCs, VSCSLCs, and VSELSCs in established cell lines. We took another look at the data we collected from HL-60 (Supplementary Figures S1 and S2) and conducted experiments on different human cell lines: AML (Figure 1), CML (Figure 2), ALL (Figure 3 and Figure S3), and Non-Small Cell Lung Cancer and Non-Cancer Embryo Kidney Cells (Figure 4 and Figure 5). These events are likely to exhibit a triple-negative status for the tested CD markers and a positive status for CD34+ (at CD45−/CD56−/CD2−) along with morphology displaying DNA with less condensation compared to ABs (Figure 1c) and are negative for 7AAD (Supplementary Figure S1). Moreover, we established the highly primitive HL-60 and highly primitive A549, in which no less than 50% of the initial culture volume is represented in scatter dot plots (Figure 5a,b). These cultures exhibited the presence of counts in regions corresponding to LSLCs, CSLCs and their progenitor-like stages, almost below 0.0%. Performing MGG staining after the Primitive HL-60 re-established enables the assessment of its proliferative potential (Figure 5c).

In future studies, it is essential to improve the effective isolation, collection, and analysis of these cellular stages, utilizing techniques, such as RNAseq and proteomics, and to focus on highlighting differences between healthy and diseased cells. Deciphering the data on cell cycle checkpoints might enhance our understanding of the molecular mechanisms behind the increased rate of malignant proliferation, as well as variations in limited cell divisions (Hayflick phenomenon) between healthy and transformed (very) small primitive stages [30,31].

## 5. Conclusions

To detect (V)SLSLCs, (V)SCSLCs, and (V)SELSCs, we analyzed the coincidence of granularity, size, and levels of CD markers confirmed to be present on VSELSCs, DNA content, and membrane integrity. However, distinguishing whether they are very small or small remains challenging. It appears likely that they are at least smaller than the previously identified LSLCs and CSLCs. Their presence suggests that they play a part in maintaining lineage continuity, and their numbers might be increased through specific cultivation conditions.

## Data Availability

The authors declare that all the imaging and biological data supporting the findings of this study are available within the paper and its Supplemental Data.

## Supplementary Materials

The following supporting information can be downloaded at: https://www.mdpi.com/article/10.3390/cancers15235520/s1, Figure S1: HL-60 cytological parameters and cluster differentiation patterns; Figure S2: Selected scatter dot-plots from gate CD45 vs. CD34, CD34 vs. CD56, and CD45 vs. CD56; Figure S3: Selected scatter dot-plots of Raji negative controls obtained during CD markers staining; Representative data; Figure S4: Selected A549 and HEK293 scatter dot-plots. Supplementary Text: ST1: Controversy; ST2: Background of Functionality Study; Supplementary References: [1–56].
