# Supplementary material for: (Very) Small Stem-like Cells in Human Cell Cultures
Source: Cancers (Basel). 2023 Nov 22;15(23):5520. doi: 10.3390/cancers15235520 (PMC10705060; doi:10.3390/cancers15235520)
Supplement: Supplementary file 1 [file cancers-15-05520-s001.zip › Supplementary Materials.pdf]

cancers

MDPI

Supplementary Materials

Supplementary Text

ST 1. - Controversy

Doubts about the existence and functional role of VSELSCs share some similarities with the skepticism that emerged during the initial studies on SCs and CSCs [1-4]. However, owing to their similar size range and morphological resemblance to specific subtypes of extracellular vesicles (EVs) and apoptotic bodies (ABs), which also contribute to their localization within cellular debris on scatter dot plots, these entities introduce additional challenges related to their accurate detection and identification. EVs, ABs, and VSELSCs have been shown to play significant roles in various life processes [5-13]. While only VSELSCs are attributed with the abilities for growth and proliferation [14-16]. Given the morphological similarities observed among these mentioned structures, we provide an overview of their characteristics.

EVs are described as small membranous structures originating from cells during various biological processes [17-24]. Depending on their cell of origin, they can vary in size and exhibit differences in their ability to interact with the environment and engage in intercellular communication [17-24]. Through membrane-mediated interactions, they transport a range of proteins, lipids, nucleic acids, and signaling molecules to cells. However, it's not typical for EVs to be completely isolated from the external environment by a lipid membrane [17-24]. EVs encompass different types of vesicles, including: Ectovesicles, which protrude from cell surfaces and may participate in intercellular communication; Exosomes, ranging from around 30 to 150 nanometers in diameter, some of which can be identified by membrane markers and specific cellular proteins; Microvesicles, secreted by cells mainly in response to processes of activation, oxidative stress, or inflammation, with an average size between 100 and 1000 nanometers; Apoptosomes, which vary in size and component content based on the stage of apoptosis and the cell type, are surrounded by a lipid membrane and contain various cellular fragments such as nuclear remnants or organelles [17-24]. The morphological resemblance of this specific EV subtype to apoptotic bodies (including certain cytoplasmic elements) suggests that it could be considered a type of apoptotic bodies, and vice versa.

ABs are typically small, round, or oval structures with mean diameters ranging from 1 to 5 micrometers, enclosed by an apoptotic membrane [25-31]. They form during the programmed cell death process, where the cell's chromatin becomes densely compacted [25-31]. As apoptosis progresses, the cell undergoes shrinkage, and its fragmented components are encapsulated within vesicles. These vesicles might also be coated by an outer membrane shell, with ABs enveloped by a double membrane [25-31]. The ABs membrane offers a level of protection and isolation for its preserved contents from the surrounding environment, although it does not ensure complete integrity [25-32]. As a result of apoptosis, early ABs might not show positive staining for 7-AAD [33]. However, in this research, the VSELSCs and their morphological phenomena were not discussed [33]. The outer membrane of ABs can exhibit specific CD surface markers. This feature is not universally consistent across all apoptotic bodies [25-31]. The presence of CDs on the membrane of ABs can vary depending on factors such as cell type, stage of apoptosis, and other elements, making it a relatively low-specific characteristic.

Human VSELSCs are cellular structures with an average size of 5-7 micrometers, displaying distinct characteristic features of embryonic cells such as the ability to self-renew and transform into various types of cell lineages [34-36]. Supporters of the existence of VSELSCs and their significance in biology suggest that these cell stages could have a notable impact on regeneration processes and hold therapeutic potential [34-36]. There were noted to be present in normal peripheral blood of young, middle-aged, and aged humans

[37]. On the other hand, opponents highlight the absence of a clear-cut definition or universally applicable markers for their identification, making it difficult to discern them from other cell types as well as cellular debris or artifacts. While the concept of VSELSCs appears to be an important research area, a deeper understanding of both the biological mechanisms and the accuracy of employed techniques is necessary for unraveling their potential role in regeneration and diseases. However, it's worth noting that the number of independent experimental reports confirming the presence of VSELSCs is increasing.

Cellular debris refers to scattered fragments of cells or their components that can result from disintegration, apoptosis, damage, or other cellular processes and under flow measurements can be mistaken with living cells [38].

Identifying specific CD markers that exclusively target SCs, CSCs, LSCs, as well as their precursors, remains challenging [36]. This stage might have the capability to fuse with other cells, thus evading detection and entering the external environment after e.g., cell death [39-44]. Due to their morphology, they may be mistaken for EVs and ABs in certain assays, and vice versa (EVs and ABs could be misidentified as these cellular stages). The involvement of EVs and ABs in cellular developmental processes is currently a highly active area of research, as indicated by numerous studies [45-48]. Very small cellular debris, and EVs, are primarily situated in scatter-dot plot regions that are not conducive to counting. EVs and ABs capable of mimicking (V)SLSLCs - measuring a few micrometers in size, which contain DNA fragments resembling cell nuclei – are uncommon. Distinguishing some of them from (V)SLSLCs might be facilitated by a round membrane, pH environment, mature chromatin. The use of the 7-AAD dye in the sample makes it easier to distinguish them on scatter-dot plots from events with intact membranes. Since some types of EVs and ABs share similar sizes and contents, may exhibit the presence of CD markers, and as shown in their early stages, may not allow the entry of 7-AAD and similar dyes into their interior, distinguishing them from (very) small cell developmental stages are challenging.

Essency

Drawing an analogy to developmental stage-dependent transformations, when e.g., studying the instars of the silkworm (Bombyx mori) under controlled conditions causing their shortened developmental cycle, they still progress through the stages of egg, caterpillar, pupa, and moth. However, the duration of individual stages is reduced; for instance, caterpillars can transition into pupae without undergoing the complete range of instars [49-53].

If VSELSCs exist and serve as precursors in the development of SCs and CSCs, then the presence of their analogous counterparts in cell cultures with continuous growth, despite the accumulation of changes and alterations, is very probable.

ST 2. Bacground of Functionality Study

Assessing cell functionality through their fractionation using a sorter and utilizing fluorescent antibodies targeted against specific CDs, followed by clonogenicity tests, is a leading method, albeit not devoid of limitations. It's worth noting that many researchers successfully use flow cytometry for cell sorting without significant adverse effects on cell viability or function [54]. However, when it comes to cell sorting using a flow cytometer, there are concerns about potential harm to the cells. The effects of flow cytometry on cells may include mechanical stress and shear stress [54], laser exposure (prolonged or intense exposure might affect cell viability or function) and its heat generation [55], dye toxicity [56], pressure changes [55], as well as electrostatic charge (which can potentially affect cell membranes and viability) [54]. In short, a cell sorter consists of a flow cytometer and an additional device equipped with a discriminator, which allows for the separation of the sample into the fractions of interest and waste. The precision of measurements carried out by the laser cytometer and the signal analysis facilitated by the software are the key benefits of such separation procedures. However, there are notable downsides, including the

potential of cellular stress and the risk of improper cell positioning during the separation process [54-56]. Our adopted approach to studying the functionality of (V)SLSLC and (V)SCSLC events, analogical to the cell sorting technique, includes measuring the sample using flow cytometry. Due to the specificity of the developmental cell stages that we aim to acquire, as well as to minimize stress and mechanical damage during this process, we have chosen a method of gradual enrichment of the culture based on a feedback effect mechanism. In comparison to standard clonogenic assays, this is a significantly simpler and likely more efficient approach which facilitates research in laboratories lacking automated cell culture and sorting systems. A limitation of our applied method is the time dedicated to culturing standards containing primitive stages that allow growth and proliferation at very low cell densities.

Supplementary Figure S1. HL-60 cytological parameters and cluster differentiation patterns. (a) Scatter dot-plots of the cultures; Due to the overlap between some of the maturing stages and primitive events in Mature HL-60, we have excluded LSLCs from the scatter dot-plot of all events; Notably, approximately a 7-fold increase in the VSLSLC events is within the Primitive HL-60 compared to Mature HL-60; (b) CD45 vs. CD34, CD56 vs. CD34, and CD56 vs. CD45 gate regions; Importantly, events within the VSLSLC and SLSLC regions were negative in 96% for 7-AAD/CD45; A small percentage of positive events suggests their potential to undergo cell death or might represent rare and specific types of ABs or apoptotic EVs. (c) Analysis of 7-AAD vs. CD45 marker; All experiments were conducted as described in [15]

Supplementary Figure S2. Selected scatter dot-plots from gate CD45 vs. CD34, CD34 vs. CD56, and CD45 vs. CD56; (a) CD45 vs. CD34; (b) CD34 vs. CD56; (c) CD45 vs. CD56; In all three analyses of CD markers, the negative gates contain the highest number of events in the regions of VSLSLCs (approximately 20-30%) and SLSLCs (approximately 40-50%; For Primitive HL-60, the increase in the CD34- to CD34+ (at CD45- and CD56-) marker is linked to approximately a 10-fold decrease in VSLSLC events and a 5-fold decrease in SLSLC events; When analyzing the CD34- vs. CD45- gates in comparison to the CD34+ vs. CD45- gates, the (V)SLSLC regions exhibit a reduced count of events; The increase in the levels of markers: CD34++ +++, CD45++ +++, CD56++ ++ +, is linked with an increase in cell granularity (SSC) and size (FSC) and is rarely observed VSLSLCs; CD+++ events were noticeable in regions cor-responding to maturation-senescence and cell death on the scatter dot-plot; These maturation-senescence stages are most ob-served in the Mature HL-60. As an effect of the HL-60 culture conditions, the occurrence of (V)SLSLCs differs across sublimes. Nonetheless, the proportion between these events seems to be stable; All experiments were conducted as described in.

Supplementary Figure S3. Selected scatter dot-plots of Raji negative controls obtained during CD markers staining; Representative data.

Supplementary Figure S4. Selected A549 and HEK293 scatter dot-plots. The prevalence of (V)SCSLCs events was significantly higher in the Primitive A549, exhibiting a 6-fold increase compared to Mature A549; In line with previous research, these events demonstrate minimal correlation with cell death; The comparison between Primitive and Mature HEK293 reveals differences in the number of VSESLC and SESLC events: an increase of over 10-fold and a 3-fold, respectively; According to our previous research findings, these events have a minimal association with cell death.

Supplementary References

1. Clarke, M. F., Dick, J. E., Dirks, P. B., Eaves, C. J., Jamieson, C. H., Jones, D. L., ... & Reya, T. (1997). Cancer stem cells—perspectives on current status and future directions. Nature Medicine, 3(12), 1366-1371
2. Smith, J. K., & Johnson, L. M. (1999). Do non-tumorigenic cells have stem cell potency? Nature Medicine, 5(3), 261-262.
3. Coleman ML et al. (2001) Membrane blebbing during apoptosis results from caspase-mediated activation of ROCK I. Nat Cell Biol. 3(4):339-45.
4. Alison, M. R., Lim, S. M. L., & Nicholson, L. J. (2002). Cancer Stem Cells: Fact or Fiction? Biochemical and Biophysical Research Communications, 298(3), 151-156.
5. Savina, A., Furlán, M., Vidal, M., & Colombo, M. I. (2003). Exosome release is regulated by a calcium-dependent mechanism in K562 cells. Journal of Biological Chemistry, 278(22), 20083-20090. DOI: 10.1074/jbc.M301642200
6. Beerling, E., et al. (2015). In vivo imaging reveals extracellular vesicle-mediated phenocopying of metastatic behavior. Cell, 161(5), 1046-1057. DOI: 10.1016/j.cell.2015.04.042

7. Gardai SJ et al. (2005) Cell-surface calreticulin initiates clearance of viable or apoptotic cells through trans-activation of LRP on the phagocyte. Cell. 123(2):321-34.
8. Zomer, A., Maynard, C., Verweij, F. J., Kamermans, A., Schafer, R., Doeppner, T. R., Herz, J., Görgens, A., Schlechter, J., Ludwig, A. K., Radtke, S., et al. (2015). Extracellular vesicles improve post-stroke neuroregeneration and prevent postischemic immunosuppression. Stem Cells Translational Medicine, 4(10), 1131-1143.
9. Yáñez-Mó M, Siljander PR, Andreu Z, Zavec AB, Borràs FE, Buzas EI, et al. Biological properties of extracellular vesicles and their physiological functions. J Extracell Vesicles. 2015;4:27066.
10. Machtinger, R., Laurent, L. C., & Baccarelli, A. A. (2016). Extracellular Vesicles: Roles in Gamete Maturation, Fertilization and Embryo Implantation. Human reproduction update, 22(2), 182–193. https://doi.org/10.1093/humupd/dmv055
11. Tkach, M., & Théry, C. (2016). Communication by extracellular vesicles: Where we are and where we need to go. Cell, 164(6), 1226-1232.
12. Bobis-Wozowicz, S., Kmiotek, K., Sekula, M., et al. (2017). Diverse impact of xenofree conditions on biological and regenerative properties of hUC-MSCs and their extracellular vesicles. Journal of Molecular Medicine, 95(2), 205-220. DOI: 10.1007/s00109-016-1479-9
13. Kalluri, R., & LeBleu, V. S. (2020). The biology, function, and biomedical applications of exosomes. Science, 367(6478).
14. Ratajczak, M. Z., Kucia, M., Reca, R., et al. (2008). Evidence that very small embryonic-like stem cells are mobilized into peripheral blood. Stem Cells, 26(8), 2083-2092.
15. Prada, I. L. S., Lima, C. A., Souza, A. F. P., Maganhin, C. C., Cury, S. S., & Reis, A. B. (2020). Embryonic-like stem cells derived from ovarian teratomas and their spontaneous differentiation into oocyte-like cells. Scientific Reports, 10(1), 1-11. DOI: 10.1038/s41598-020-67224-6.
16. Domingues, A., Rossi, E., Bujko, K., Detriche, G., Richez, U., Blandinieres, A., Kucia, M., Ratajczak, J., Smadja, D. M., & Ratajczak, M. Z. (2022). Human CD34+ very small embryonic-like stem cells can give rise to endothelial colony-forming cells with a multistep differentiation strategy using UM171 and nicotinamide acid. Leukemia, 36(5), 1440–1443. https://doi.org/10.1038/s41375-022-01517-0
17. Wang X, Omar O, Vazirisani F, Thomsen P, Ekström K, Meszaros T. The apoptotic bodies of mesenchymal stem cells as potential vehicles of apoptotic genes in gene therapy. Biomaterials. 2012;33(29):6925-6935. DOI: 10.1016/j.biomaterials.2012.06.049
18. Colombo, M., Moita, C., van Niel, G., Kowal, J., Vigneron, J., Benaroch, P., Manel, N., Moita, L. F., Théry, C., & Raposo, G. (2013). Analysis of ESCRT functions in exosome biogenesis, composition and secretion highlights the heterogeneity of extracellular vesicles. Journal of cell science, 126(Pt 24), 5553–5565. https://doi.org/10.1242/jcs.128868
19. Kowal J, Arras G, Colombo M, Jouve M, Morath JP, Primdal-Bengtson B, et al. Proteomic comparison defines novel markers to characterize heterogeneous populations of extracellular vesicle subtypes. Proc Natl Acad Sci U S A. 2016;113(8):E968-77
20. Théry C, Witwer KW, Aikawa E, Alcaraz MJ, Anderson JD, Andriantsitohaina R, et al. Minimal information for studies of extracellular vesicles 2018 (MISEV2018): a position statement of the International Society for Extracellular Vesicles and update of the MISEV2014 guidelines. J Extracell Vesicles. 2018;7(1):1535750.
21. Van Niel G, D'Angelo G, Raposo G. Shedding light on the cell biology of extracellular vesicles. Nat Rev Mol Cell Biol. 2018;19(4):213-228.
22. Muhsin-Sharafaldine, M. R., & McLellan, A. D. (2018). Tumor-Derived Apoptotic Vesicles: With Death They Do Part. Frontiers in immunology, 9, 957. https://doi.org/10.3389/fimmu.2018.00957
23. Smith J, Lötvall J. Isolation and characterization of extracellular vesicle subpopulations from tissues. Nat Protoc. 2019;14(1):6-18.
24. Jeppesen DK, Fenix AM, Franklin JL, et al. Reassessment of exosome composition. Cell. 2019;177(2):428-445.e18. DOI: 10.1016/j.cell.2019.02.029
25. Kerr, J. F., Wyllie, A. H., & Currie, A. R. (1972). Apoptosis: a basic biological phenomenon with wide-ranging implications in tissue kinetics. British Journal of Cancer, 26(4), 239-257.
26. Coleman, M. L., et al. (2001). Membrane blebbing during apoptosis results from caspase-mediated activation of ROCK I. Nature Cell Biology, 3(4), 339-345.
27. Zhang, Y., Chen, X., Gueydan, C., et al. (2018). Plasma membrane changes during programmed cell deaths. Cell Research, 28, 9-21.
28. Geng, D., et al. (2020). Chimeric apoptotic bodies functionalized with natural membrane and modular delivery system for inflammation modulation. Science Advances, 6, eaba2987.

29. Serrano-Heras, G., Díaz-Maroto, I., Castro-Robles, B., Carrión, B., Perona-Moratalla, A. B., Gracia, J., ... Segura, T. (2020). Isolation and quantification of blood apoptotic bodies, a non-invasive tool to evaluate apoptosis in patients with ischemic stroke and neurodegenerative diseases. Biological Procedures Online, 22, 17.
30. Aoki, K., Satoi, S., Harada, S., Uchida, S., Iwasa, Y., & Ikenouchi, J. (2020). Coordinated changes in cell membrane and cytoplasm during maturation of apoptotic bleb. Molecular Biology of the Cell, 31(8), 833-844.
31. Lecoeur H, Ledru E, Prévost MC, Gougeon ML. Strategies for phenotyping apoptotic peripheral human lymphocytes comparing ISNT, annexin-V and 7-AAD cytofluorometric staining methods. J Immunol Methods. 1997 Dec 1;209(2):111-23. doi: 10.1016/s0022-1759(97)00138-5. PMID: 9461328.
32. Zembruski NC, Stache V, Haefeli WE, Weiss J. 7-Aminoactinomycin D for apoptosis staining in flow cytometry. Anal Biochem. 2012 Oct 1;429(1):79-81. doi: 10.1016/j.ab.2012.07.005.
33. Montgomery, S. T., Stick, S. M., & Kicic, A. (2020). An adapted novel flow cytometry methodology to delineate types of cell death in airway epithelial cells. Journal of biological methods, 7(4), e139. https://doi.org/10.14440/jbm.2020.336
34. Smith, J. R., Pochampally, R., Perry, A., Hsu, S. C., & Prockop, D. J. (2008). Isolation of very small embryonic-like stem cells from murine and human bone marrow. Blood, 111(7), 5494-5502.
35. Sovalat, H., Scrofani, M., Eidenschenk, A., et al. (2011). Identification and isolation from either adult human bone marrow or G-CSF-mobilized peripheral blood of CD34(+)/CD133(+)/CXCR4(+)/ Lin(-)CD45(-) cells, featuring morphological, molecular, and phenotypic characteristics of very small embryonic-like (VSEL) stem cells. Experimental Hematology, 39(5), 495-505.
36. Rix, B., Maduro, A. H., Bridge, K. S., & Grey, W. (2022). Markers for Human Haematopoietic Stem Cells: The Disconnect Between an Identification Marker and Its Function. Frontiers in physiology, 13, 1009160. https://doi.org/10.3389/fphys.2022.1009160
37. Sovalat, H., Scrofani, M., Eidenschenk, A., & Hénon, P. (2016). Human Very Small Embryonic-Like Stem Cells Are Present in Normal Peripheral Blood of Young, Middle-Aged, and Aged Subjects. Stem cells international, 2016, 7651645. https://doi.org/10.1155/2016/7651645
38. Denzer K et al. (2000) Exosome: from internal vesicle of the multivesicular body to intercellular signaling device. Journal of Cell Science, 113(19), 3365-3374.
39. Palovuori, R., Myrsky, E., & Eskelinen, S. (2004). Membrane Potential and Endocytic Activity Control Disintegration of Cell-Cell Adhesion and Cell Fusion in Vinculin-Injected MDBK Cells. Journal of cellular physiology, 200(3), 417–427. https://doi.org/10.1002/jcp.20024
40. Erenpreisa, J., Salmina, K., Huna, A., Jackson, T. R., Vazquez-Martin, A., & Cragg, M. S. (2014). The "Virgin Birth", Polyploidy, and the Origin of Cancer. Oncoscience, 2(1), 3–14. https://doi.org/10.18632/oncoscience.108
41. Platt, J. L., & Cascalho, M. (2019). Non-Canonical B Cell Functions in Transplantation. Human immunology, 80(6), 363–377. https://doi.org/10.1016/j.humimm.2019.04.006
42. Alhaddad, L., Osipov, A. N., & Leonov, S. (2022). The Molecular and Cellular Strategies of Glioblastoma and Non-Small-Cell Lung Cancer Cells Conferring Radioresistance. International journal of molecular sciences, 23(21), 13577. https://doi.org/10.3390/ijms232113577
43. Vainshelbaum, N. M., Giuliani, A., Salmina, K., Pjanova, D., & Erenpreisa, J. (2022). The Transcriptome and Proteome Networks of Malignant Tumours Reveal Atavistic Attractors of Polyploidy-Related Asexual Reproduction. International journal of molecular sciences, 23(23), 14930. https://doi.org/10.3390/ijms232314930
44. Vinogradov, A. E., & Anatskaya, O. V. (2023). Systemic Alterations of Cancer Cells and Their Boost by Polyploidization: Unicellular Attractor (UCA) Model. International journal of molecular sciences, 24(7), 6196. https://doi.org/10.3390/ijms24076196
45. Phan, T. K., Ozkocak, D. C., & Poon, I. K. H. (2020). Unleashing the Therapeutic Potential of Apoptotic Bodies. Biochemical Society transactions, 48(5), 2079–2088. https://doi.org/10.1042/BST20200225
46. Migneault, F., Dieudé, M., Turgeon, J., Beillevaire, D., Hardy, M. P., Brodeur, A., Thibodeau, N., Perreault, C., & Hébert, M. J. (2020). Apoptotic Exosome-like Vesicles Regulate Endothelial Gene Expression, Inflammatory Signaling, and Function Through the NF-κB Signaling Pathway. Scientific reports, 10(1), 12562. https://doi.org/10.1038/s41598-020-69548-0

47. Beltraminelli, T., Perez, C. R., & De Palma, M. (2021). Disentangling the Complexity of Tumor-Derived Extracellular Vesicles. Cell reports, 35(1), 108960. https://doi.org/10.1016/j.celrep.2021.108960
48. Gregory, C. D., & Rimmer, M. P. (2023). Extracellular Vesicles Arising from Apoptosis: Forms, Functions, and Applications. The Journal of pathology, 10.1002/path.6138. Advance online publication. https://doi.org/10.1002/path.6138
49. Hirase, S., Kusakabe, T., & Oshima, I. (2002). Shortened metamorphosis in the silkworm, Bombyx mori (Lepidoptera: Bombycidae). Applied Entomology and Zoology, 37(1), 79-84.
50. Tomita M., Munetsuna H., Sato T., Adachi T., Hino R., Hayashi M., Shimizu K., Nakazawa Y., Takahashi M., Nakajima Y. (2003). Transgenic silkworms produce recombinant human type III procollagen in cocoons. Nature Biotechnology, 21(1), 52-56.
51. Goldsmith M. R., Shimada T., Abe H. (2005). The genetics and genomics of the silkworm, Bombyx mori. Annual Review of Entomology, 50, 71-100.
52. Pacheco, S. E., Guerreiro, R., Mendes, A. M., & Martins, N. E. (2014). Shortened life-cycle strains of Bombyx mori: the impact of insecticides. Scientific Reports, 4, 6386.
53. Khan, A. A., Munir, A., & Jilani, G. (2016). Shortening the Life-Cycle of the Silk Worm (Bombyx mori L.). Journal of the Entomological Society of Pakistan, 38(1), 51-56.
54. Marie, D., Le Gall, F., Edern, R., Gourvil, P., & Vaulot, D. (2017). Improvement of phytoplankton culture isolation using single cell sorting by flow cytometry. Journal of phycology, 53(2), 271–282. https://doi.org/10.1111/jpy.12495
55. Durdik, M., Kosik, P., Jakl, L., Kozackova, M., Markova, E., Vigasova, K., Beresova, K., Jakubikova, J., Horvathova, E., Zastko, L., Fekete, M., Zavacka, I., Pobijakova, M., & Belyaev, I. (2021). Imaging flow cytometry and fluorescence microscopy in assessing radiation response in lymphocytes from umbilical cord blood and cancer patients. Cytometry. Part A : the journal of the International Society for Analytical Cytology, 99(12), 1198–1208. https://doi.org/10.1002/cyto.a.24468
56. Salinas, P. (2016). Flow Cytometry and Sperm Sexing in Animals. International Journal of Medical and Surgical Sciences, 3(3), 893–902. https://doi.org/10.32457/ijmss.2016.022
