# Supplementary figures and images for: (Very) Small Stem-like Cells in Human Cell Cultures
Source: Cancers (Basel). 2023 Nov 22;15(23):5520. doi: 10.3390/cancers15235520 (PMC10705060; doi:10.3390/cancers15235520)

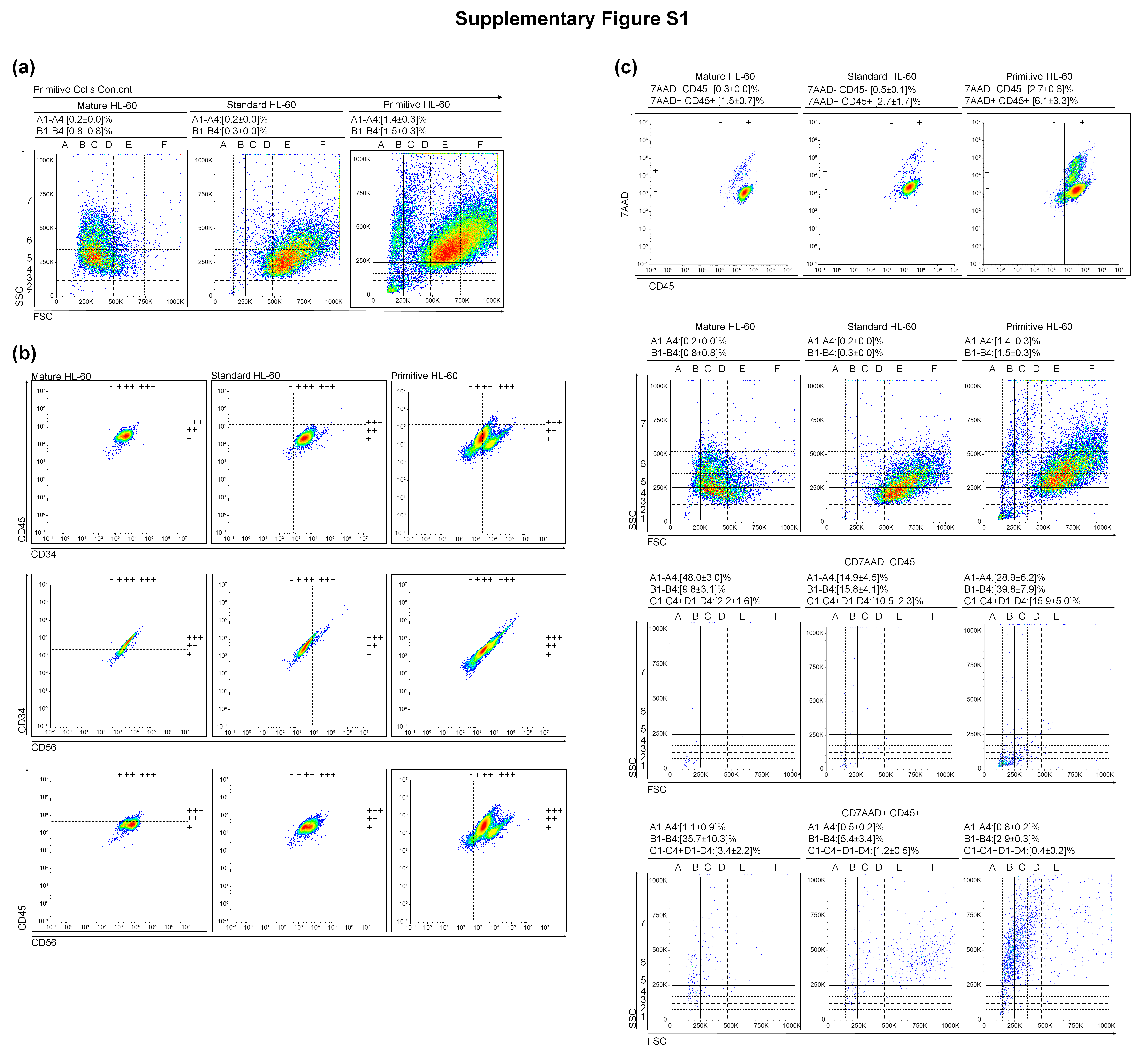

Supplement: Supplementary file 1 [file cancers-15-05520-s001.zip › Supplementary Figure S1.png]

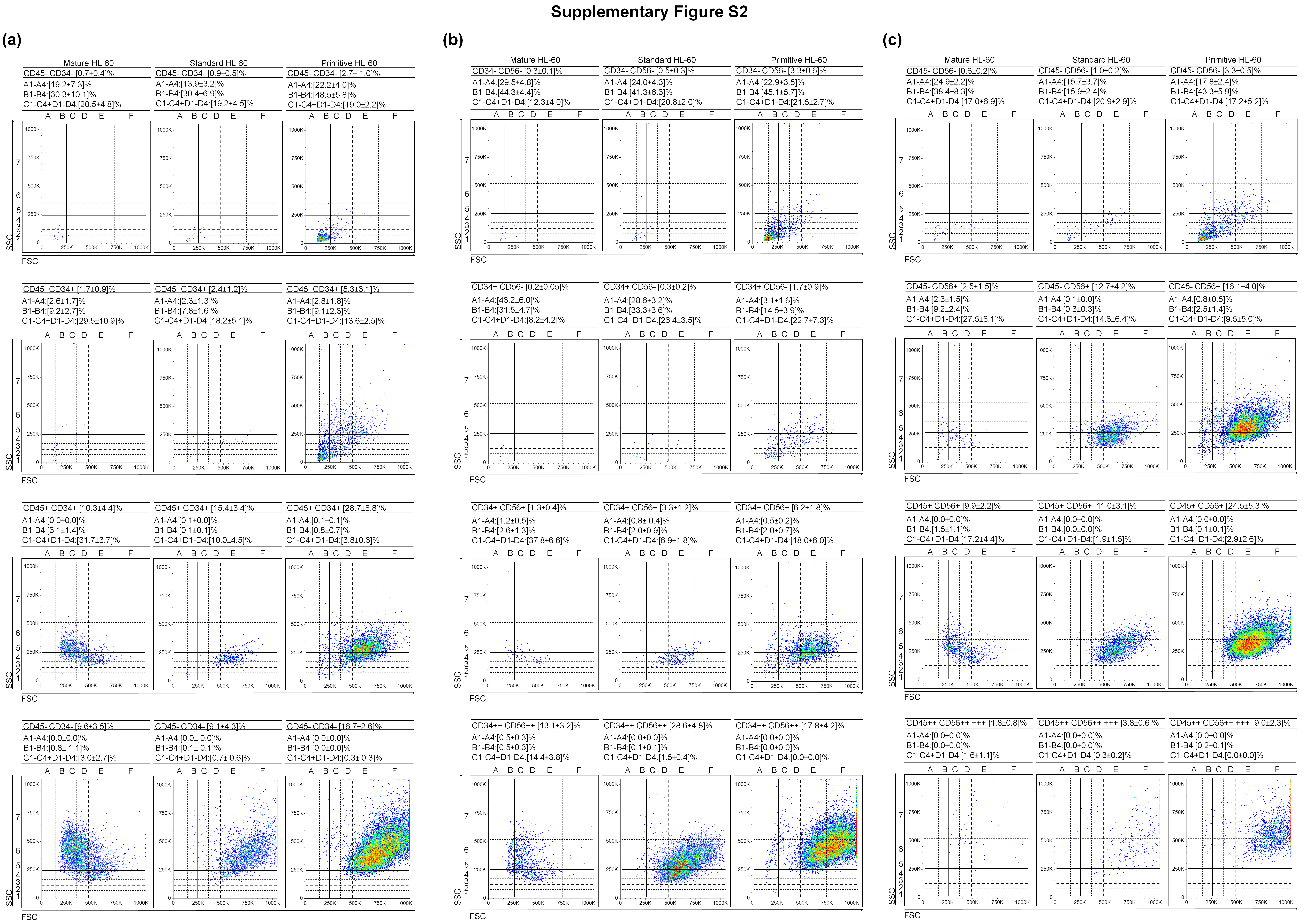

Supplement: Supplementary file 1 [file cancers-15-05520-s001.zip › Supplementary Figure S2.png]

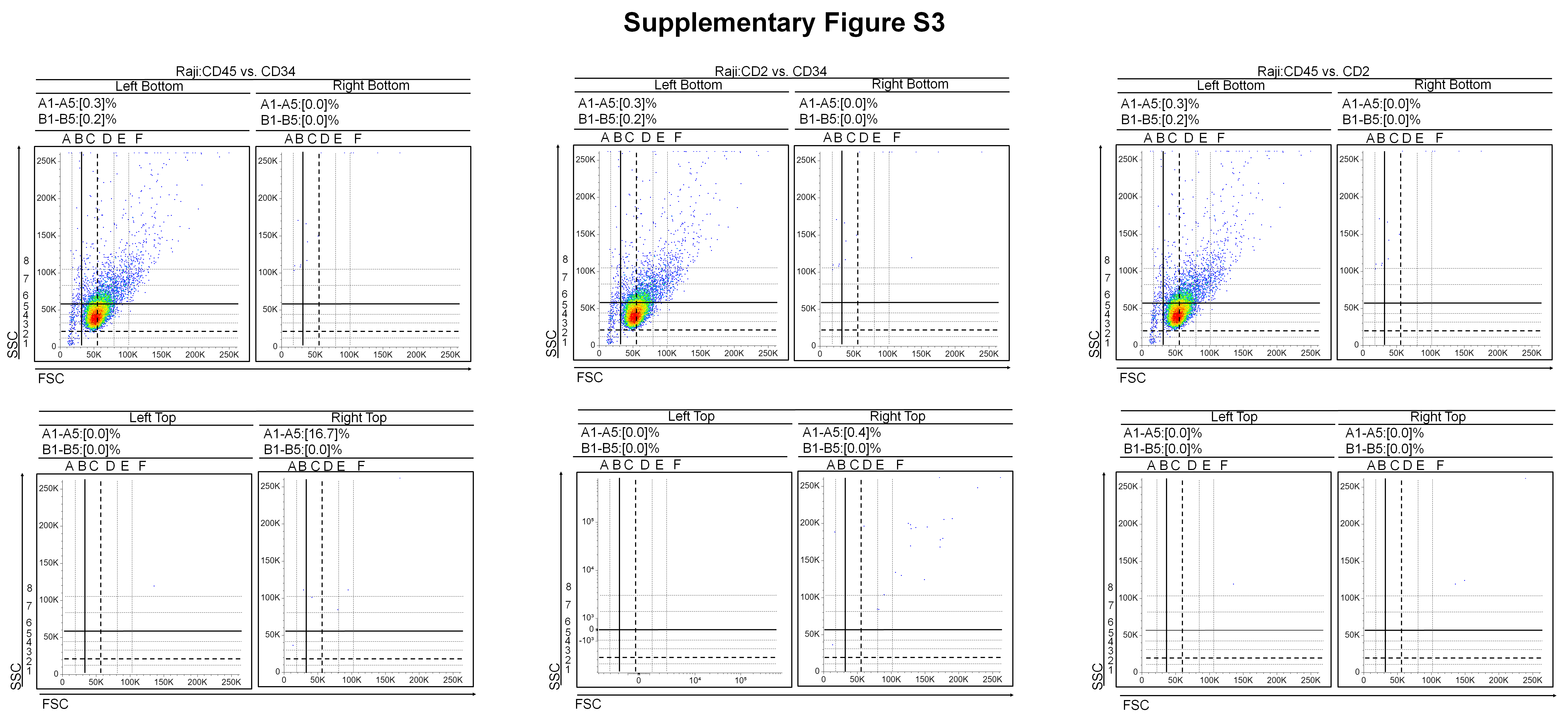

Supplement: Supplementary file 1 [file cancers-15-05520-s001.zip › Supplementary Figure S3.png]

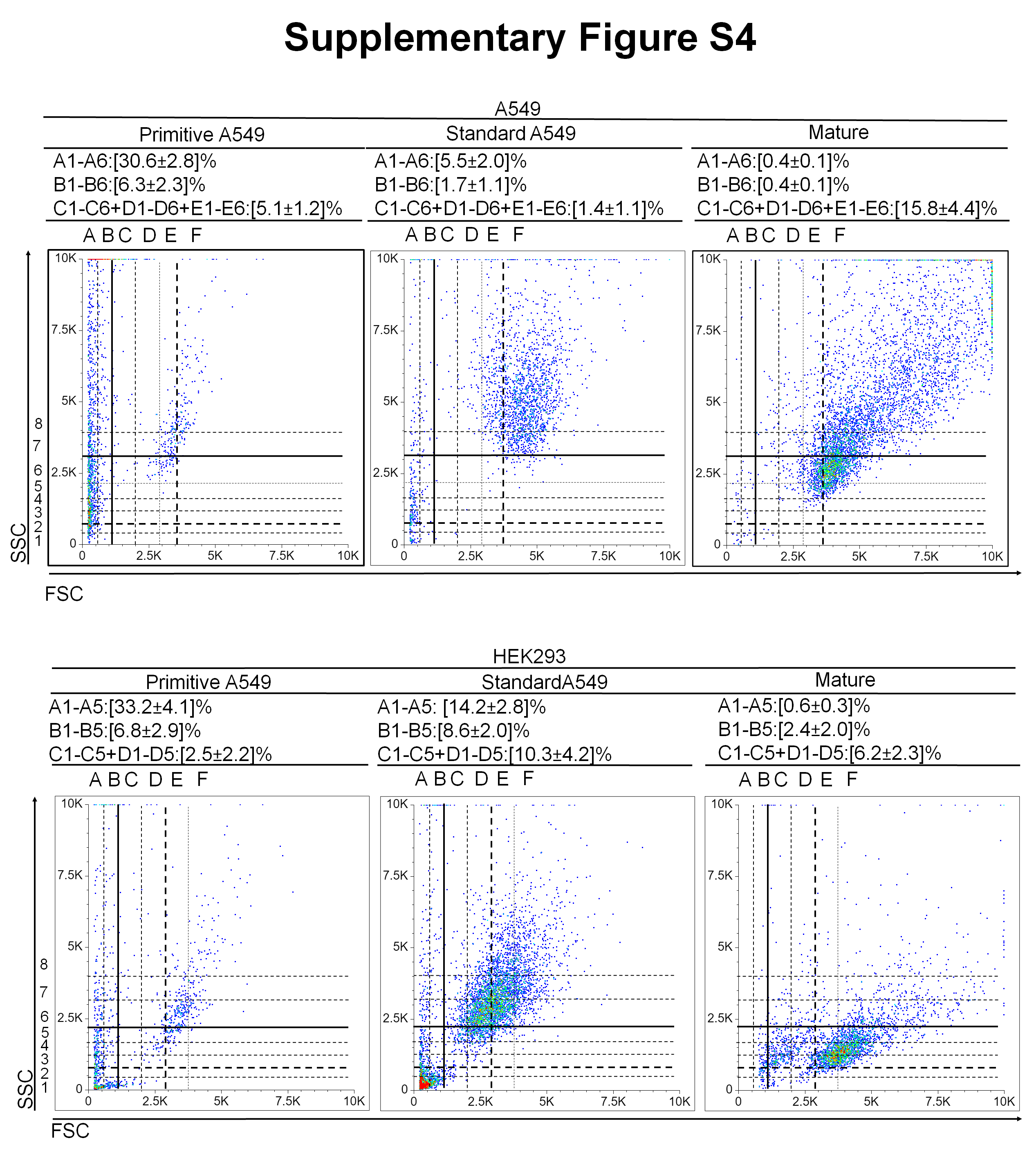

Supplement: Supplementary file 1 [file cancers-15-05520-s001.zip › Supplementary Figure S4.png]
